# Concurrent Activation of Liver X Receptor and Peroxisome Proliferator-Activated Receptor Alpha Exacerbates Hepatic Steatosis in High Fat Diet-Induced Obese Mice

**DOI:** 10.1371/journal.pone.0065641

**Published:** 2013-06-07

**Authors:** Mingming Gao, Le Bu, Yongjie Ma, Dexi Liu

**Affiliations:** Department of Pharmaceutical and Biomedical Sciences, College of Pharmacy, University of Georgia, Athens, Georgia, United States of America; National Institute of Agronomic Research, France

## Abstract

Liver X receptor (LXR) activation improves glucose homeostasis in obesity. This improvement, however, is associated with several side effects including hyperlipidemia and hepatic steatosis. Activation of peroxisome proliferator-activated receptor alpha (PPARα), on the other hand, increases fatty acid oxidation, leading to a reduction of hyperlipidemia. The objective of this study was to investigate whether concurrent activation of LXR/PPARα can produce synergistic benefits in treating obesity-associated metabolic disorders. Treatment of high fat diet-induced obese mice with T0901317, an LXR activator, or fenofibrate, the PPARα agonist, or in combination alleviated insulin resistance and improved glucose tolerance. The combined treatment dramatically exacerbated hepatic steatosis. Gene expression analysis in the liver showed that combined treatment increased the expression of genes involved in lipogenesis and fatty acid transport, including *srebp-1c*, *chrebp*, *acc1*, *fas*, *scd1* and *cd36*. Histochemistry and *ex vivo* glycerol releasing assay showed that combined treatment accelerated lipid mobilization in adipose tissue. Combined treatment also increased the transcription of *glut4*, *hsl*, *atgl* and *adiponectin*, and decreased that of *plin1*, *cd11c*, *ifnγ* and *leptin*. Combined treatment markedly elevated the transcription of *fgf21* in liver but not in adipose tissue. These results suggest that concurrent activation of LXR and PPARα as a strategy to control glucose and lipid metabolism in obesity is beneficial but could lead to elevation of lipid accumulation in the liver.

## Introduction

Liver X receptors (LXR) are transcription factors belonging to the nuclear receptor superfamily. Since their initial discovery in 1995, LXRs have emerged as powerful metabolic regulators in different tissues and cell types. LXRs have been shown to regulate cholesterol, bile acid, triglyceride and glucose homeostasis as well as inflammation and intestinal lipid absorption [1]. Similar to LXR, peroxisome proliferator-activated receptor alpha (PPARα) is a ligand-activated transcription factor that belongs to the steroid hormone receptor superfamily. PPARα is expressed predominantly in tissues that have a high level of fatty acid catabolism, such as the liver, heart, and muscle [2]. PPARα regulates the expression of a number of genes critical for lipid and lipoprotein metabolism, and PPARα ligand fibrates are used for the treatment of dyslipidemia due to their ability to lower plasma triglyceride levels and elevate HDL cholesterol levels. Physiologically, both LXR and PPARα need to form heterodimers with retinoid X receptor (RXR) to initiate the expression of their target genes [3]. Therefore, a tight cross-talk exists between LXR and PPARα [4], [5].

LXR activation produces a variety of beneficial effects in managing metabolic disorders. For example, previous studies by Cao *et al.* and Laffitte *et al.* show that LXR activation improves glucose tolerance in diabetic animal models [6], [7]. Consistent with these studies, our recent work demonstrates that activation of LXR protects mice from high fat diet-induced obesity and insulin resistance [8]. In addition, murine studies have shown positive effects of LXR agonists on insulin resistance and atherosclerosis [9], [10]. Due to these beneficial effects, LXR has been identified as attractive pharmacological target for management of metabolic disorders. Unfortunately, these beneficial effects are associated with several severe side effects including hyperlipidemia and hepatic steatosis [8], [11].

On the other hand, activation of PPARα accelerates lipid absorption and increases fatty acid oxidation, leading to an improvement in lipid metabolism and a reduction of hyperlipidemia [12], [13], [14]. Moreover, PPARα activators have been shown to regulate obesity in rodents by both increasing hepatic fatty acid oxidation and decreasing levels of circulating triglycerides responsible for adipose cell hypertrophy and hyperplasia [15], [16].

The focus of the current study is to assess the effects of concurrent activation of LXR and PPARα on systemic metabolism and hepatic fat accumulation under the status of obesity in which the metabolism of glucose and lipids are dysregulated. We demonstrate that combined treatment by T0901317, a potent activator of LXR, and fenofibrate, an agonist of PPARα, alleviated insulin resistance and improved glucose tolerance. Surprisingly, this combined treatment dramatically exacerbated hepatic steatosis in obese mice. Mechanistic studies suggest the exacerbation effect is caused by increased lipogenesis in the liver and accelerated lipid mobilization in the adipose tissue.

## Methods

### Ethics Statement

The use of animals in this study was in compliance with relevant federal guidelines and institutional policies and the animal protocol was approved by the IACUC of the University of Georgia.

### Animals and Animal Treatments

Male C57BL/6 mice (23–25 g, Charles River, Wilmington, MA, USA) were fed a high fat diet (Bio-serv, F3282) for 12 weeks and became obese. These mice were then divided into four groups (5 each), including the control group, a T0901317-treated group, a fenofibrate-treated group and a group with a combined treatment of T0901317 and fenofibrate (Cayman Chemical, Ann Arbor, MI, USA). Mice in the control group were injected with carrier solution (dimethyl sulfoxide), and mice in treated groups were injected with T0901317 (2.5 mg/kg/day, *i.p.*), fenofibrate (25 mg/kg/day, *i.p.*) or a combination of T0901317 and fenofibrate (T0901317, 2.5 mg/kg/day; fenofibrate, 25 mg/kg/day, *i.p.*), respectively. The injections were performed daily for 5 days while keeping animals on high fat diet. After the last injection, mice were fasted overnight and sacrificed using carbon dioxide.

### Intraperitoneal Glucose Tolerance Test (IPGTT) and Insulin Tolerance Test (ITT)

IPGTT and ITT were performed using 4 groups of obese mice (5 each) treated with the same regimen. Mice employed in IPGTT were fasted for 6 h before the test. Glucose solubilized in phosphate buffered saline was injected (*i.p.*) at 1.5 g/kg, and the time-point was set as 0 min. Blood glucose levels at 0, 30, 60 and 120 min were measured using glucose meters and glucose test strips. Mice utilized in ITT were fasted for 4 h before the injection of insulin (Humulin, 0.75 U/kg) from Eli Lilly (Indianapolis, IN), and blood glucose levels were measured at 0, 30, 60 and 120 min after insulin injection.

### Histochemical Study

For haematoxylin and eosin (H&E) staining, tissues of interest were dissected and fixed overnight in 10% neutral buffered formalin. The samples were dehydrated using gradient ethanol and embedded in paraffin. Tissue sections were cut at 6 µm in thickness and dried at 37°C for 2 h. H&E staining were performed using a commercial kit (#3500, BBC Biochemical). For the frozen section and Oil-red O staining, liver samples were frozen in liquid nitrogen and sectioned at 8 µm in thickness using a Cryostat. These sections were placed on slides and washed with 60% isopropanol before being stained with Oil-red O (Electron Microscopy Sciences) for 30 min and counterstained using haematoxylin. The tissue slides were examined using Nikon ECLIPSE-Ti optical microscope and pictures were taken and analyzed using Nikon NIS-Elements AR software.

### Liver Triglyceride Assay

Liver triglyceride levels were determined following a previously reported method [17], [18]. Briefly, liver samples (200–400 mg) were homogenized in a mixture of chloroform and methanol (2∶1, volume ratio). The homogenates were kept at 4°C overnight before being centrifuged at 12,000 rpm for 20 min. Supernatants were transferred into new tubes, dried and then dissolved in 5% Triton-X100. The triglyceride concentration was determined using a commercial kit (#TR22203, Thermo-Scientific).

### Biochemical Analysis of Blood Samples

Blood samples were collected from animals which were fasted overnight and centrifuged at 3,000 rpm for 10 min to isolate plasma. Plasma concentrations of glucose (Thermo-Scientific), insulin (Mercodia), triglyceride (Thermo-Scientific), cholesterol (Genzyme Diagnostics) and free fatty acids (BioAssay Systems) were determined using commercial kits. Based on fasting glucose and fasting insulin levels, we also calculated the value of Homeostasis Model of Assessment - Insulin Resistance (HOMA-IR) using the following formula: HOMA-IR = [fasting insulin (ng/mL) × fasting plasma glucose (mg/dL)/405] [19].

### Gene Expression Analysis

Liver and white adipose tissue samples were freshly collected and immediately frozen at −80°C until use. Total RNA was purified using TRIZOL reagent from Invitrogen (Grand Island, NY). The purified RNA samples were dissolved in RNase-free water and kept at −80°C and quantitative real-time PCR (qPCR) was performed using SYBR Green as detection reagent. The primers for qPCR were synthesized at Sigma (St. Louis, MO) and their sequences are listed in the Table S1. For all primers, melting curve analysis was conducted and confirmed as a single DNA duplex. The ΔΔCt method was employed for quantitative examination and the GAPDH mRNA was used as an internal control [20].

### *Ex vivo* Glycerol Releasing Assay

The *ex vivo* glycerol releasing assay was conducted following a previously reported method [21]. Briefly, epididymal white adipose tissue was dissected and cut into small pieces (∼20 mg per piece). The cut tissues were put into Krebs-Ringer buffer (12 mM HEPES, 121 mM NaCl, 4.9 mM KCl, 1.2 mM MgSO_4_ and 0.33 mM CaCl_2_) with 3.5% fatty acid-free BSA and 0.1% glucose. Supernatants were collected at pre-determined time-points and their glycerol levels were measured using a commercial kit purchased from Cayman Chemical (Ann Arbor, MI).

### Statistical Analysis

Statistical analysis was performed using one-way analysis of variance. The results were expressed as the mean ± SD. A *p* value below 0.05 (*p*<0.05) was considered significantly different.

## Results

### Combined Treatment by T0901317 and Fenofibrate Exacerbated Hepatic Steatosis in High Fat Diet-induced Obese Mice

**Figure 1:**
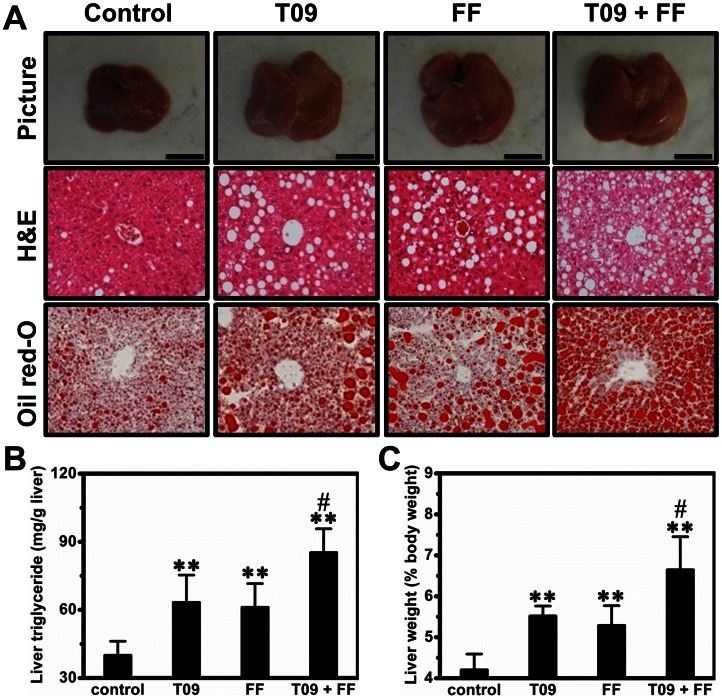
Effect of T0901317 (T09), finofibrate (FF) or in combination (T09+ FF) on hepatic steatosis in high fat diet-induced obese mice. (A) Representative pictures and histochemistry of the liver. *Upper panel*, pictures of the liver (bar length = 1 cm); *middle panel*, results of H&E staining; *lower panel*, results of Oil-red O staining. (B) Effect of combined treatment on triglyceride levels in the liver. (C) Effect of combined treatment increased liver weight. Values presented in (C) – (D) are average ± SD (n = 5); **p<0.01 *vs* control; ^#^ *p*<0.05 *vs* T0901317-treated animals.

Compared to control and animals treated with either T0901317 or fenofibrate, combined treatment greatly enlarged liver size, which could be recognized even with the naked eye (Figure 1A). The order of the number of vacuoles seen by H&E staining is: combination group> T0901317-treated group ≈ fenofibrate-treated group>> control group (Figure 1A), suggesting that combined treatment significantly aggravated liver fat accumulation. A similar trend is evident by Oil-red O staining (Figure 1A). To define the degree of hepatic steatosis among these animals, we extracted lipids from the livers and measured their triglyceride content. Activation of LXR, PPARα or both increased the liver triglyceride level from 40.0±6.2 (mg/g tissue) in control animals to 63.3±11.9, 61.1±10.5, and 85.3±10.5 (mg/g tissue), respectively (Figure 1B). The liver triglyceride level with the combined treatment was significantly higher compared to that of control group (*p*<0.01) and T0901317-treated group (*p*<0.05), suggesting that combined treatment had a synergistic effect on hepatic steatosis in high fat diet-induced obese mice. We also measured liver weight (Figure 1C) and observed a similar trend: the mouse livers received combined treatment weighs ∼6.6% of body weight, greater than ∼5.5% of body weight for T0901317-treated animals ≈ that of fenofibrate-treated animals (∼5.3% of body weight). The average liver weight for control animals was approximately 4.2% of body weight of the obese mice. Additionally, we repeated this experiment using additional 4 groups of obese mice (5 each) without performing overnight fasting, and obtained similar results showing that combined treatment markedly exacerbated hepatic steatosis (data not shown).

### Impact of LXR or/and PPARα Activation on Glucose and Lipid Levels in the Blood

**Figure 2:**
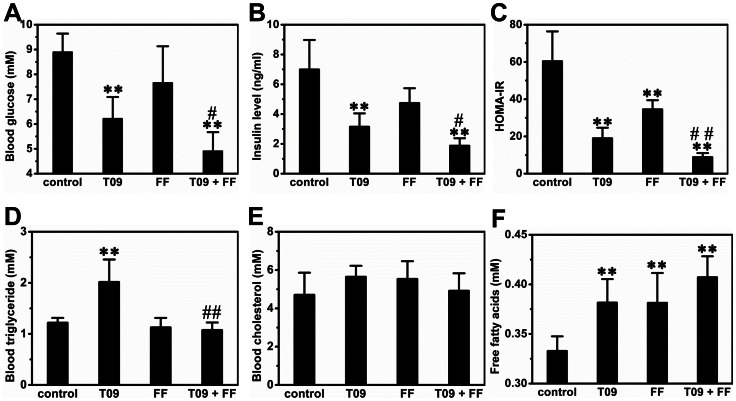
Effect of T0901317 (T09), finofibrate (FF) or in combination (T09+ FF) on blood levels of insulin, glucose, triacylglyceride, cholesterol, and free fatty acid. (A) Blood glucose level. (B) Blood insulin level. (C) Results of HOMA-IR. (D) Blood triglyceride level. (E) Blood cholesterol level. (F) Blood free fatty acids level. Values represent average ± SD (n = 5); **p<0.01 *vs* control; ^#^ *p*<0.05 *vs* T0901317-treated animals; ^##^ *p*<0.01 *vs* T0901317-treated animals.

To assess the impact of different treatments on glucose homeostasis, we measured fasting glucose and insulin levels. Figure 2A shows that T0901317 treatment reduced blood glucose concentration from 8.9±0.7 to 6.2±0.8 mM (*p*<0.01). Less impact was seen in animals treated with fenofibrate (∼7.5 mM). The most significant impact was seen in animals who received the combined treatment reaching blood concentration at 4.9±0.7 mM (*p*<0.05 *vs* T0901317-treated group). Similarly, the combined treatment has the highest impact on insulin level with fasting insulin concentration of 1.9±0.5 ng/ml, comparing to 7.0±2.0 ng/ml in control animals, and 3.2±0.9 ng/ml and 4.7±1.0 ng/ml in animals treated with T0901317 or fenofibrate, respectively (*p*<0.01, Figure 2B), suggesting that the insulin resistance was greatly improved by the combined treatment. The HOMA-IR value, which reflects the degree of insulin resistance, showed ∼85% reduction in animals with combined treatment (Figure 2C). Regarding blood levels of triglyceride, cholesterol and free fatty acids, T0901317 treatment increased blood triglyceride by ∼1.7-fold and combined treatment completely blocked the elevation (Figure 2D). There was no difference in cholesterol level (Figure 2E) among animals either treated or untreated. All treated animals showed a higher level of free fatty acids in the blood (Figure 2F).

### Combined Treatment by T0901317 and Fenofibrate Improved Glucose Tolerance and Alleviated Insulin Resistance

**Figure 3:**
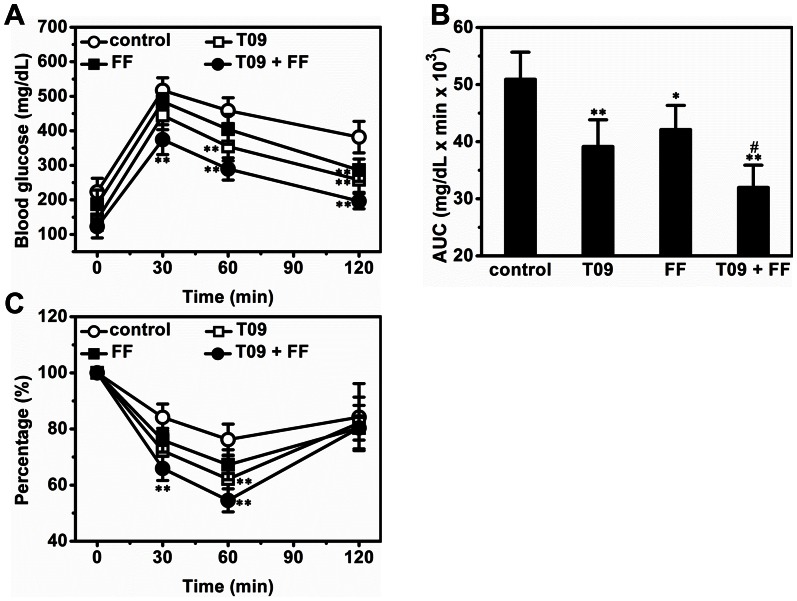
Effect of T0901317 (T09), finofibrate (FF) or in combination (T09+ FF) on glucose tolerance and insulin sensitivity. (A) Glucose profiles of IPGTT. (B) Area under the curve (AUC) of IPGTT. (C) Results of ITT. Values represent average ± SD (n = 5); *p<0.05 *vs* control; **p<0.01 *vs* control; ^#^ *p*<0.05 *vs* T0901317-treated animals.

To further investigate the impact of combined treatment on glucose homeostasis, we performed IPGTT. Combined treatment markedly improved the glucose tolerance of obese mice in IPGTT, reducing the glucose peak level by ∼27.6% compared to control (Figure 3A). AUC calculations make this point clearer (Figure 3B). Next we conducted ITT to assess the impact of combined treatment on insulin resistance. As expected, the insulin resistance of obese mice was greatly alleviated by combined treatment (Figure 3C).

### Impact on Gene Expression in the Liver

**Figure 4:**
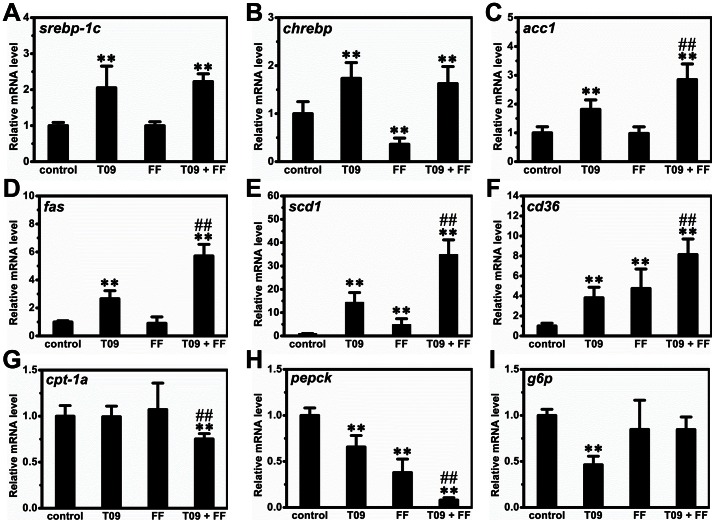
Effect of T0901317 (T09), finofibrate (FF) or in combination (T09+ FF) on gene expression. (A) – (E) The mRNA level of the genes involved in lipogenesis (*srebp-1c*, *chrebp*, *acc1*, *fas,* and *scd1)*. (F) – (G) The mRNA level of genes involved in fatty acid transport and β-oxidation (*cd36*, *cpt-1a)*. (H) - (I) The mRNA level of genes involved in gluconeogenesis (*pepck* and *g6p).* Values represent average ± SD (n = 4–5); **p<0.01 *vs* control; ^##^ *p*<0.01 *vs* T0901317-treated animals.

**Figure 5:**
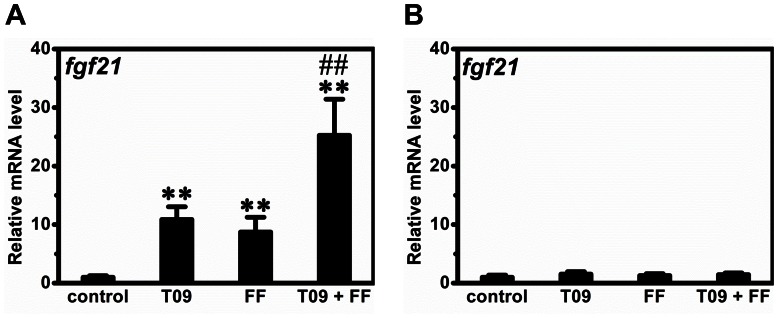
Effect of T0901317 (T09), finofibrate (FF) or in combination (T09+ FF) on ***fgf21*** **gene expression in liver and adipose tissue.** (A) The mRNA level of *fgf21* in the liver. (B) The mRNA level of *fgf21* in adipose tissue. Values represent average ± SD (n = 4); **p<0.01 *vs* control; ^##^ *p*<0.01 *vs* T0901317-treated animals.

To confirm the activation of these nuclear receptors, we selected a set of target genes and measured their mRNA levels. As expected, T0901317 treatment greatly increased mRNA levels of *cyp7a1*, *abcg5* and *abcg8*, 3 target genes of LXR, by ∼4.3-fold, ∼3.5-fold and ∼4.1-fold respectively (Figures S1A–C). Similarly, fenofibrate treatment markedly elevated mRNA levels of *cyp4a10* and *cyp4a14*, 2 target genes of PPARα, by ∼29.8-fold and ∼32.1-fold, respectively (Figures S1D–E). To explore the possible mechanism, we measured the expression of genes involved in lipid and glucose metabolism in the liver. The *srebp-1c* and *chrebp* are two master genes for lipogenesis in the liver. Combined treatment significantly increased the expression of *srebp-1c* and *chrebp* by 2.2±0.2-fold and 1.6±0.4-fold, respectively (Figures 4A and 4B), indicating that this treatment increased lipogenesis. We also measured the expression of *acc1*, *fas* and *scd1*, 3 vital genes involving lipogenesis, and found that the combined treatment increased their expression by 2.9±0.5, 5.7±0.8 and 34.8±6.3 folds, respectively (Figures 4C, 4D and 4E). Collectively, these data suggest that combined treatment enhanced lipogenesis in the liver. Combined treatment also increased transcription of the *cd36* gene encoding fatty acid transporter by 8.2±1.5 folds (Figure 4F). The expression of *cpt1a* coding for CPT1a for fatty acid β-oxidation was significantly reduced by the combined treatment (Figure 4G). In addition, combined treatment decreased the expression of *pepck* but not *g6p* (Figures 4H and 4I), two crucial genes for gluconeogenesis in the liver. To further explore the regulation network, we measured mRNA levels of several nuclear receptors involved in lipid metabolism including *lxr*α, *ppar*α, *ppar*γ and *ppar*δ. Combined treatment did not significantly change mRNA levels of *ppar*α, *ppar*α and *ppar*δ while greatly reducing that of *ppar*γ (Figures S1F–I). Additionally, we determined the transcription of *fgf21* whose expression is regulated by both PPARα and LXR. As expected, activation of either PPARα or LXR markedly increased the transcription of *fgf21* (Figure 5A) in the liver but the increase was much higher with combined treatment. However, none of the treatments affects *fgf21* gene expression in the adipose tissue (Figure 5B).

### Combined Treatment Reduced Adipocyte Size

**Figure 6:**
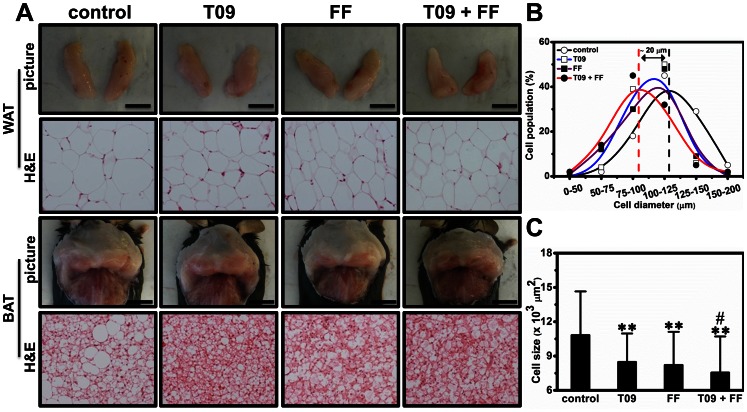
Effect of T0901317 (T09), finofibrate (FF) or in combination (T09+ FF) on adipocyte size. (A) Images of adipose tissue (bar length = 1 cm) and sections of adipose tissue. (B) Percentage of cells with defined range of diameters. (C) Average size of adipocytes. **p<0.01 *vs* control; ^#^ *p*<0.05 *vs* T0901317-treated animals.

Combined treatment reduced the size of epididymal white adipose tissue (WAT) (Figure 6A), indicating this treatment accelerated lipid mobilization in WAT. Interestingly, combined treatment also reduced the size of white adipocytes surrounding intrascapular brown adipose tissue (BAT) (Figure 6A). Similarly, combined treatment decreased the number and size of vacuolar structures in BAT as indicated by the white areas of the tissue section with H&E staining (Figure 6A), suggesting that this treatment also reduced fat deposited in BAT. We measured the diameter of white adipocytes and found combined treatment significantly reduced their diameter by ∼20 µm (Figure 6B) and size (Figure 6C), further indicating that the treatment stimulated lipid mobilization in WAT (*p*<0.01 *vs* control; *p*<0.05 *vs* T0901317-treated group).

### Combined Treatment Accelerated Lipid Mobilization in WAT

**Figure 7:**
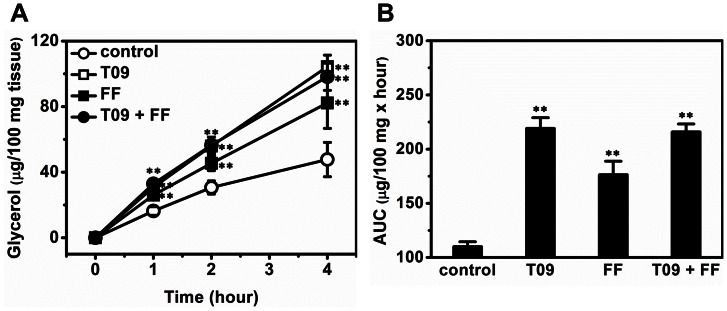
Effect of T0901317 (T09), finofibrate (FF) or in combination (T09+ FF) on glycerol release from WAT. (A) Time course of the glycerol release. (B) AUC of the glycerol release. Values represent average ± SD (n = 3); **p<0.01 *vs* control.

A glycerol releasing assay was employed to confirm the effect of combined treatment on lipid mobilization in WAT. Results in Figure 7A show that the glycerol release rate in *ex-vivo* was ∼15.8, ∼33.8, ∼26.6, and ∼31.8 (µg/100 mg tissue/hour) for control, T0901317-treated, fenofibrate-treated animals, and animals with combined treatment, respectively. Similarly, the value of area under curve (AUC) also shows combined treatment increased glycerol releasing by ∼2 folds (Figure 7B).

### Gene Expression in WAT

**Figure 8:**
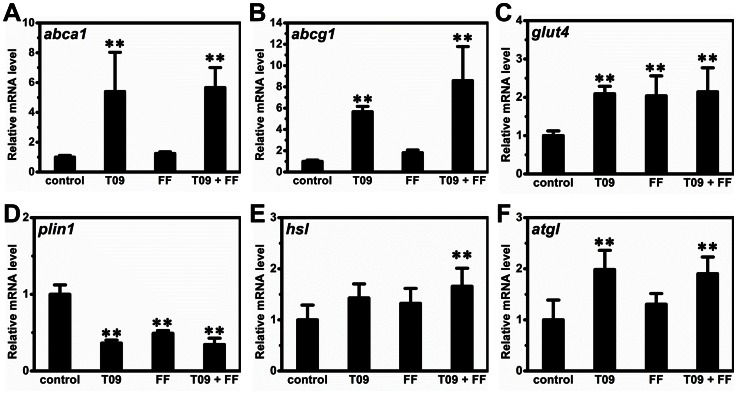
Effect of T0901317 (T09), finofibrate (FF) or in combination (T09+ FF) on mRNA levels of selected genes in the adipose tissue. (A) – (B) The mRNA levels of genes involved in cholesterol reverse transport (*abca1* and *abcg1)*. (C) The mRNA level of *glut4 gene*. (D) The mRNA level of *plin1 gene*. (E) – (F) The mRNA level of *hsl* and *atgl genes*. **p<0.01 *vs* control.

**Figure 9:**
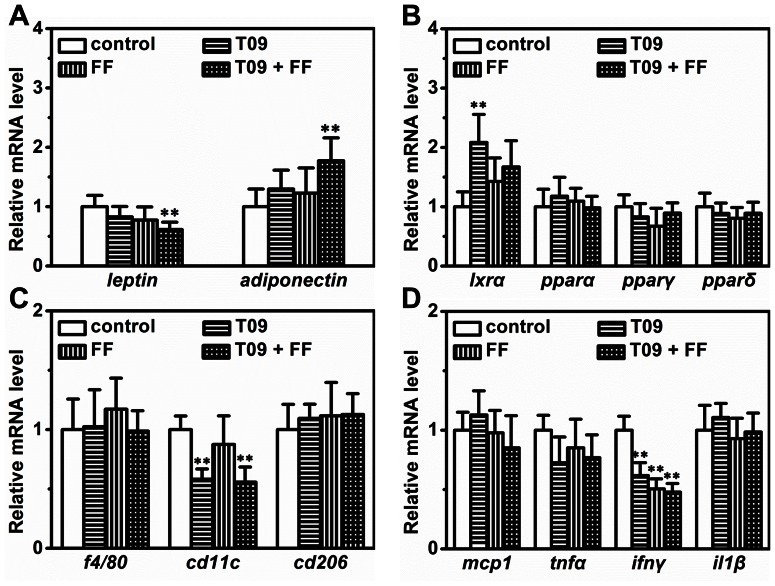
The mRNA level of selected genes in adipose tissue. (A) The mRNA level of *leptin* and *adiponectin* genes. (B) The mRNA level of *lxrα*, *pparα*, *pparγ* and *pparδ*. (C) The mRNA level of *f4/80*, *cd11c* and *cd206*. (D) The mRNA level of *mcp1*, *tnfα*, *ifnγ* and *il1β* Values represent average ± SD (n = 4); **p<0.01 *vs* control.

To investigate the impact of combined treatment on the transcription of genes involved in lipid and glucose transport and storage, including *abca1*, *abcg1*, *glut4* and *plin1*, we measured their mRNA levels in WAT. *Abca1* and *abcg1* are vital for cholesterol reverse transport, and both are target genes of LXR. As expected, combined treatment significantly increased the expression of these two genes (Figures 8A and 8B). *Glut4* is pivotal for intracellular transport of glucose and its expression level is tightly correlated with insulin sensitivity. Combined treatment increased the transcription of the *glut4* gene (Figure 8C). Expression of *plin1* gene encoding a protein that coats lipid droplets and protects lipids against lipolysis was greatly decreased in animals treated with T0901317, fenofibrate or in combination (Figure 8D). Meanwhile, mRNA levels of *hsl* and *atgl* were slightly but significantly increased by combined treatment (Figures 8E and 8F). We also determined mRNA levels of several adipokines including *fgf21*, *leptin* and *adiponectin*. Surprisingly, the transcription of *fgf21* in adipose tissue was not significantly changed (Figure 5). Combined treatment slightly down-regulated the transcription of *leptin* and up-regulated that of *adiponectin* (Figure 9A). Interestingly, T0901317 increased mRNA level of *lxrα* without significant change on that of *pparα*, *pparγ* and *pparδ* (Figure 9B). In addition, combined treatment suppressed gene expression of *cd11c* and *ifnγ* that are tightly correlated with the severity of chronic inflammation (Figures 9C–D).

## Discussion

In this study, we demonstrate that activation of LXR by T0901317, PPARα by fenofibrate or in combination, has significant impact on glucose and lipid homeostasis and lipid accumulation in the liver and adipose tissues of high fat diet-induced obese mice. Concurrent activation of both nuclear receptors dramatically exacerbated hepatic steatosis in high fat diet-induced obese mice (Figure 1) but alleviated insulin resistance, improved glucose tolerance and blocked T0901317-induced hyperlipidemia (Figures 2 and 3). In the liver, combined treatment decreased the transcription of genes for gluconeogenesis and increased mRNA level of genes for lipogenesis and fatty acid transport (Figure 4). In WAT the treatment increased the expression of genes for cholesterol and glucose transport and decreased the transcription of *plin1* which protects lipid droplets from lipolysis (Figure 8). Combined treatment accelerated lipid mobilization in WAT, as evidenced by reduced size of white adipocytes and enhanced glycerol release (Figures 6 and 7).

**Figure 10:**
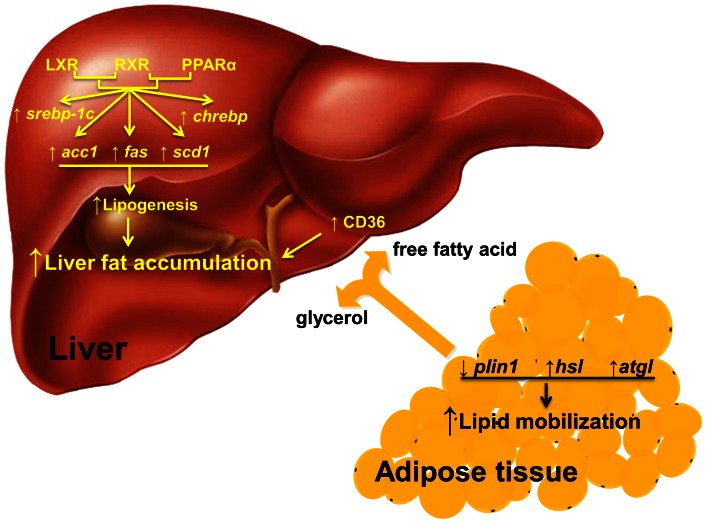
Depicted pathway for the exacerbated hepatic steatosis caused by combined treatment. Combined treatment activates LXR and PPARα, thereby increasing lipogenesis in the liver via elevating the transcription of *srebp-1c*, *chrebp*, *acc1*, *fas* and *scd1*. Meanwhile combined treatment accelerates lipid mobilization in the adipose tissue, thereby releasing more free fatty acids that are transported into hepatocytes by CD36 and further aggravate hepatic steatosis.

The exacerbation effect of combined treatment could be attributed to increased lipogenesis in the liver and accelerated lipid mobilization in WAT, as depicted in Figure 10. Physiologically, activated LXR or PPARα forms heterodimer with retinoid X receptor (RXR) and initiates the transcription of different sets of genes for cholesterol reverse transport and fatty acid metabolism, respectively [3]. Both nuclear receptors are involved in lipogenesis in the liver [22]. Activation of both LXR and PPARα significantly increased lipogenesis via elevating the transcription of *srebp-1c*, *chrebp*, *acc1*, *fas* and *scd1* (Figure 4). Meanwhile, combined treatment accelerated lipid mobilization in WAT and released more free fatty acids into the circulation (Figures 2F, 6 and 7). The fatty acids entered hepatocytes primarily via CD36 whose transcription was up-regulated by the combined treatment (Figure 3F). Collectively, the endogenously generated and exogenously acquired lipids aggregate in the liver and exacerbate hepatic steatosis in these obese mice.

Combined treatment by activators of LXR and PPARα exerted a synergistic effect in increasing lipogenesis in the liver via elevating the transcription of *acc1*, *fas* and *scd1*. Consistent with previous studies by Grefhorst *et al.* and Chisholm *et al.* showing that LXR activation leads to hepatic steatosis [11], [23], our data also show LXR activation significantly increased the expression of genes involved in lipogenesis, including *srebp-1c*, *chrebp*, *acc1*, *fas* and *scd1* (Figure 3), leading to elevated blood triglyceride and aggravated hepatic steatosis. Oosterveer *et al.* reported PPARα activation using fenofibrate simultaneously induces fatty acid oxidation and synthesis in the liver, resulting in increased hepatic triglycerides [24]. On the other hand, Larter *et al.* showed activation of PPARα by Wy14643, another agonist of PPARα, improved hepatic steatosis in female diabetic *foz/foz* mice [25]. Coinciding with the results of Oosterveer *et al.*, our data also show fenofibrate increased the transcription of *scd1* and aggravated liver fat accumulation (Figure 3E). The discrepancy between our results and that of Larter *et al.* is likely resulted from the two different animal models employed, one employing wild type HFD-induced obese mice and the other using *Alms1* mutant (*foz/foz*) mice [25], [26]. Interestingly, simultaneous activation of LXR and PPARα by T0901317 and fenofibrate exerted a synergistic effect in elevating the transcription of *acc1*, *fas* and *scd1* (Figures 3C to 3E), three key genes in the pathway of lipogenesis. This synergistic effect may play an important role in causing the aggravated hepatic steatosis in obese mice.

In agreement with the study by Stenson *et al.* showing LXR activation enhanced lipolysis *in vitro* [27], our data demonstrate that LXR activation accelerated lipid mobilization and glycerol release in WAT *in vivo* and *ex vivo* (Figures 6 and 7). The impact of LXR activation on lipid turnover in WAT is complicated. Based on the results of cell culture *in vitro*, some studies proposed LXR activation enhances lipid accumulation in adipocytes [28], [29], [30], but others did not [27], [31]. In line with the study by Stenson *et al.* and Ross *et al.* [27], [31], our data clearly show LXR activation by T0901317 accelerated lipid mobilization and glycerol release *in vivo* and *ex vivo*, probably via coordinately modulating the expression of *plin1*, *hsl* and *atgl* (Figures 8D–F). In addition, since insulin is the primary hormone controlling fat storage in adipocytes, the reduced level of blood insulin (Figure 2B) achieved by combined treatment may also contribute to lipolysis in adipose tissue. Besides WAT, BAT plays critical roles in lipid metabolism as well [32]. Interestingly, our histochemical examinations show LXR activation decreased fat accumulation in BAT (Figure 6A), which is different from previous study by Korach-André *et al.* showing LXR deficiency decreased BAT lipid accumulation in LXR gene knockout mice [33]. The discrepancy between these two studies is likely resulted from the two different animal models employed, one employing wild type obese mice and the other using LXR gene knockout mice. Alternatively, this discrepancy could be that the reduced BAT fat observed in this study is a consequence of systemic activation of LXR rather than a direct local effect. Collectively, this accelerated lipid mobilization in adipose tissue may contribute in exacerbating hepatic steatosis in the high fat diet-induced obese mice.

Accumulating evidence suggests the existence of a tight cross-talk between LXR and PPARα in multiple tissues [4], [5], [34], [35], [36]. In response to endogenous and exogenous signals, both LXR and PPARα heterodimerizes with RXR to initiate the expression of the target genes. Therefore, RXR is also involved in this network. For example, a previous study by Lenhard *et al.* showed that activation of RXR by LG100268, a potent agonist of this nuclear receptor, decreased blood glucose but markedly increased hepatic fat accumulation in *db*/*db* mice [37]. Using luciferase reporter gene assay and gel shift assay, Yamada and colleagues elegantly demonstrated that LXR-RXR-PPARα forms a network that tightly regulates lipid degradation and lipogenesis [4], [5]. Subsequent investigations by Colin *et al.* and Ducheix *et al.* provide valuable information for this cross-talk *in vivo*, showing LXR-PPARα drives lipid metabolism in response to oxysterol and fatty acids [34], [35]. Using chromatin immunoprecipitation-sequencing, Boergesen *et al.* further elucidated the extensive cross-talk between LXR and PPARα at the level of binding to shared genomic sites [36]. Consistent with these studies, our data show that simultaneous activation of LXR/PPARα synergistically modulated the expression of a set of genes involved in lipid and glucose metabolism in liver, including *acc1*, *fas*, *scd1*, *cd36* and *pepck* (Figure 4). Similar to these genes, *fgf21* is also regulated by LXR and PPARα [38], [39], [40]. Recently, Dutchak *et al.* demonstrated that FGF21 is also an inducible autocrine factor in adipose tissue that functions to regulate PPARγ [41]. Interestingly, our data show that simultaneous activation of LXR/PPARα greatly increased the transcription of *fgf21* in liver but not in adipose tissue (Figure 5), indicating that different regulatory mechanism may exist in these two tissues. In addition, because T0901317 may activate other nuclear receptors besides LXR [42], further studies are required to better elucidate this cross-talk at molecular level.

Macrophage infiltration and chronic inflammation are features of diet-induced obesity and contribute to development of obesity-associated metabolic disorders including glucose intolerance and fatty liver [43], [44]. LXR activation has a well-defined role in suppressing inflammation, and activators of LXR are negative regulators of macrophage inflammatory gene expression [45], [46]. Similar to that of LXR, PPARα activation produces an anti-inflammatory effect as well [47]. Emerging evidence suggests that LXR and PPARα signaling influences multiple facets of inflammation and immunity, thereby providing important cross-talk between metabolism and immune system [48]. In this context, we determined the transcription of a set of genes involved in chronic inflammation, and found that simultaneous activation of LXR/PPARα slightly but significantly reduced mRNA levels of *cd11c* and *ifnγ* (Figure 9). This reduction may also contribute to the improvement in insulin resistance and glucose tolerance.

In summary, in this study we demonstrate that combined treatment by activators of LXR and PPARα alleviated insulin resistance and improved glucose homeostasis but dramatically exacerbated hepatic steatosis in high fat diet-induced obesity. This exacerbation effect is caused by increased lipogenesis in the liver and accelerated lipid mobilization in WAT. Collectively, these data suggest that cautions should be taken in considering activation of both LXR and PPARα as a strategy for treatment of obesity or obesity related diseases.

## Supporting Information

Figure S1 **Effect of T0901317 (T09), finofibrate (FF) or in combination (T09+ FF) on the mRNA level of selected genes in the liver.** (A) – (C) The mRNA levels of LXR target genes including *cyp7a1*, *abcg5* and *abcg8*. (D) – (E) The mRNA level of PPARα target genes including *cyp4a10* and *cyp4a14.* (F) – (C) The mRNA level of a set of nuclear receptor genes including *lxrα*, *pparα*, *pparγ* and *pparδ*. Values represent average ± SD (n = 4); **p<0.01 *vs* control.
(TIF)

Table S1 **Primer sequences for gene expression analysis.**
(DOC)
