# Supplementary figures and images for: Concurrent Activation of Liver X Receptor and Peroxisome Proliferator-Activated Receptor Alpha Exacerbates Hepatic Steatosis in High Fat Diet-Induced Obese Mice
Source: PLoS One. 2013 Jun 7;8(6):e65641. doi: 10.1371/journal.pone.0065641 (PMC3676322; doi:10.1371/journal.pone.0065641)

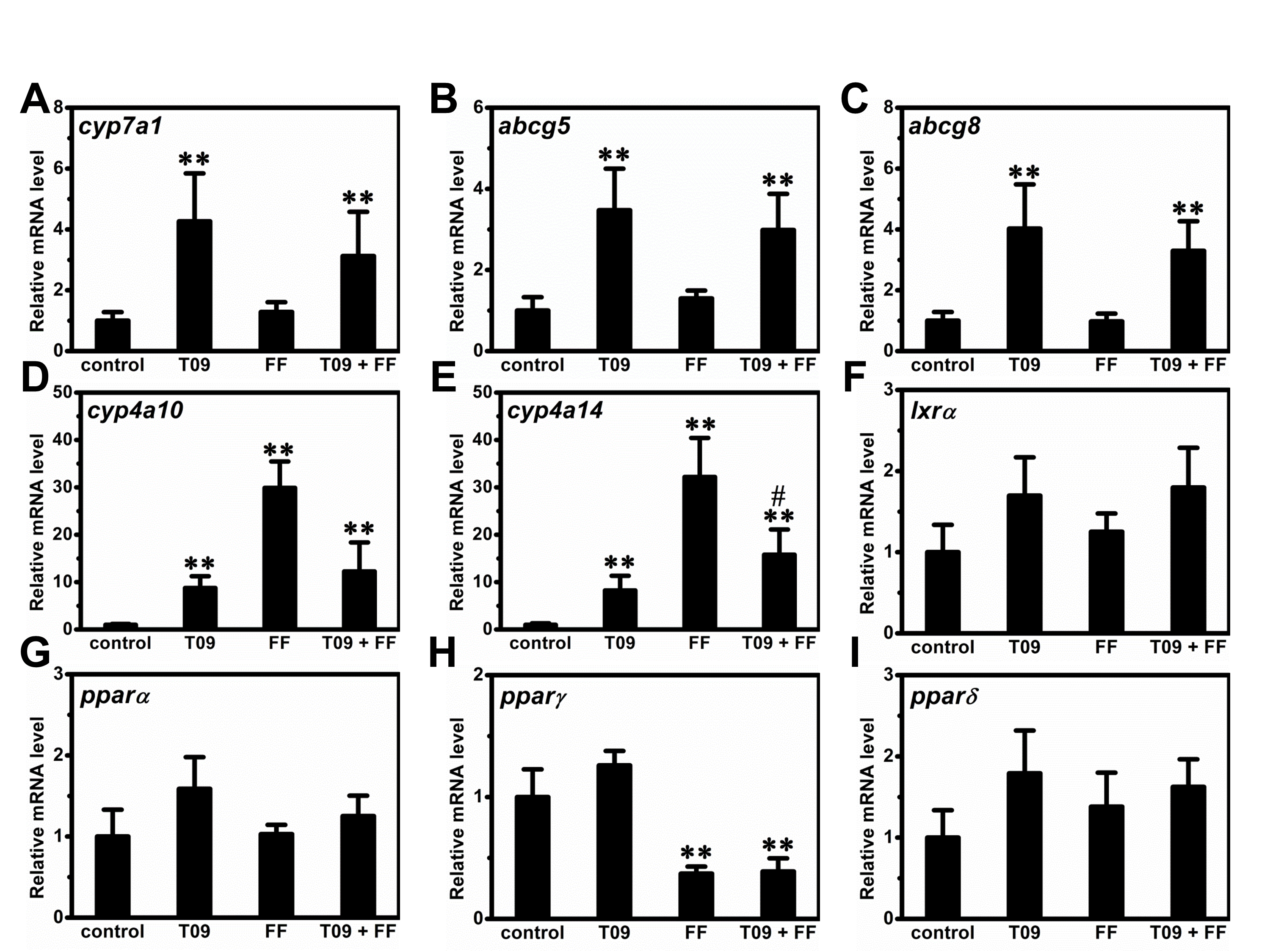

Supplement: Figure S1 — Effect of T0901317 (T09), finofibrate (FF) or in combination (T09+ FF) on the mRNA level of selected genes in the liver. (A) – (C) The mRNA levels of LXR target genes including cyp7a1, abcg5 and abcg8. (D) – (E) The mRNA level of PPARα target genes including cyp4a10 and cyp4a14. (F) – (C) The mRNA level of a set of nuclear receptor genes including lxrα, pparα, pparγ and pparδ. Values represent average ± SD (n = 4); **p<0.01 vs control. (TIF) [file pone.0065641.s001.tif]
