# Supplementary material for: Concurrent Activation of Liver X Receptor and Peroxisome Proliferator-Activated Receptor Alpha Exacerbates Hepatic Steatosis in High Fat Diet-Induced Obese Mice
Source: PLoS One. 2013 Jun 7;8(6):e65641. doi: 10.1371/journal.pone.0065641 (PMC3676322; doi:10.1371/journal.pone.0065641)
Supplement: Table S1 — Primer sequences for gene expression analysis. (DOC) [file pone.0065641.s002.doc]

**Table S1. Primer sequences for gene expression analysis**

| **Gene name** | **Primer** |
| --- | --- |
| *Abca1* | F: ATGGG CGGAATGGTCTCTTTC  R: GTAGCTCAGGCGTACAGAGAT |
| *Abcg1* | F: CTTTCCTACTCTGTACCCGAGG  R: CGGGGCATTCCATTGATAAGG |
| *Abcg5* | F: AGGGCCTCACATCAACAGAG  R: GCTGACGCTGTAGGACACAT |
| *Abcg8* | F: CTGTGGAATGGGACTGTACTTC  R: TTGGACTGACCACTGTAGGT |
| *Acc1* | F: ATGGG CGGAATGGTCTCTTTC  R: TGGGGACCTTGTCTTCATCAT |
| *Adiponectin* | F: TGTTCCTCTTAATCCTGCCCA  R: CCAACCTGCACAAGTTCCCTT |
| *Cd11c* | F: CTGGATAGCCTTTCTTCTGCTG  R: GCACACTGTGTCCGAACTCA |
| *Cd206* | F: CTCTGTTCAGCTATTGGACGC  R: CGGAATTTCTGGGATTCAGCTTC |
| *Cd36* | F: ATGGGCTGTGATCGGAACTG  R: GTCTTCCCAATAAGCATGTCTCC |
| *Chrebp* | F: AGATGGAGAACCGACGTATCA  R: ACTGAGCGTGCTGACAAGTC |
| *Cpt-1a* | F: CTCCGCCTGAGCCATGAAG  R: CACCAGTGATGATGCCATTCT |
| *Cyp7a1* | F: GGGATTGCTGTGGTAGTGAGC  R: GGTATGGAATCAACCCGTTGTC |
| *Cyp4a10* | F: TTCCCTGATGGACGCTCTTTA  R: GCAAACCTGGAAGGGTCAAAC |
| *Cyp4a14* | F: TTTAGCCCTACAAGGTACTTGGA  R: GCAGCCACTGCCTTCGTAA |
| *F4/80* | F: TGACTCACCTTGTGGTCCTAA  R: CTTCCCAGAATCCAGTCTTTCC |
| *Fas* | F: GGAGGTGGTG ATAGCCGGTAT  R: TGGGTAATCCATAGAGCCCAG |
| *Fgf21* | F: CTGCTGGGGGTCTACCAAG  R: CTGCGCCTACCACTGTTCC |
| *Glut4* | F: GTGACTGGAACACTGGTCCTA  R: CCAGCCACGTTGCATTGTAG |
| *G6p* | F: CGACTCGCTATCTCCAAGTGA  R: GTTGAACCAGTCTCCGACCA |
| *Ifnγ* | F: ATGAACGCTACACACTGCATC  R: CCATCCTTTTGCCAGTTCCTC |
| *Il1β* | F: GCAACTGTTCCTGAACTCAACT  R: ATCTTTTGGGGTCCGTCAACT |
| *Leptin* | F: GAGACCCCTGTGTCGGTTC  R: CTGCGTGTGTGAAATGTCATTG |
| *Lxrα* | F: CTCAATGCCTGATGTTTCTCCT  R: TCCAACCCTATCCCTAAAGCAA |
| *Mcp1* | F: TTAAAAACCTGGATCGGAACCAA  R: GCATTAGCTTCAGATTTACGGGT |
| *Pepck* | F: CTGCATAACGGTCTGGACTTC  R: CAGCAACTGCCCGTACTCC |
| *Plin1* | F: GGGACCTGTGAGTGCTTCC  R: GTATTGAAGAGCCGGGATCTTTT |
| *Pparα* | F: AGAGCCCCATCTGTCCTCTC  R: ACTGGTAGTCTGCAAAACCAAA |
| *Pparγ* | F: TCGCTGATGCACTGCCTATG  R: GAGAGGTCCACAGAGCTGATT |
| *Pparδ* | F: TCCATCGTCAACAAAGACGGG  R: ACTTGGGCTCAATGATGTCAC |
| *Scd1* | F: TTCTTGCGATACACTCTGGTGC  R: CGGGATTGAATGTTCTTGTCGT |
| *Srebp-1c* | F: GCAGCCACCATCTAGCCTG  R: CAGCAGTGAGTCTGCCTTGAT |
| *Tnfα* | F: CCCTCACACTCAGATCATCTTCT  R: GCTACGACGTGGGCTACAG |
